# What COVID-19 Reveals about the Neglect of WASH within Infection Prevention in Low-Resource Healthcare Facilities

**DOI:** 10.4269/ajtmh.20-0638

**Published:** 2020-09-28

**Authors:** Joanne A. McGriff, Lindsay Denny

**Affiliations:** The Center for Global Safe, Water, Sanitation and Hygiene, Rollins School of Public Health, Emory University, Atlanta, Georgia

## Abstract

The highly infectious nature of the SARS-CoV-2 virus requires rigorous infection prevention and control (IPC) to reduce the transmission of COVID-19 within healthcare facilities, but in low-resource settings, the lack of water access creates a perfect storm for low-handwashing adherence, ineffective surface decontamination, and other environmental cleaning functions that are critical for IPC compliance. Data from the WHO/UNICEF Joint Monitoring Programme show that one in four healthcare facilities globally lacks a functional water source on premises (i.e., basic water service); in sub-Saharan Africa, half of all healthcare facilities have no basic water services. But even these data do not tell the whole story, other water, sanitation, and hygiene (WASH) assessments in low-resource healthcare facilities have shown the detrimental effects of seasonal or temporary water shortages, nonfunctional water infrastructure, and fluctuating water quality. The rapid spread of COVID-19 forces us to reexamine prevailing norms within national health systems around the importance of WASH for quality of health care, the prioritization of WASH in healthcare facility investments, and the need for focused, cross-sector leadership and collaboration between WASH and health professionals. What COVID-19 reveals about infection prevention in low-resource healthcare facilities is that we can no longer afford to “work around” WASH deficiencies. Basic WASH services are a fundamental prerequisite to compliance with the principles of IPC that are necessary to protect patients and healthcare workers in every setting.

As a global pandemic, COVID-19 has caught us unprepared. As of September 2020, more than 27 million people have been infected and nearly 900,000 lives have been lost worldwide within the span of months.^1^ Even worse, many of our public health and hospital systems in high-income countries where the outbreak started, like the United States and Italy, are failing to meet the immense challenges of diagnostic testing, contact tracing, and high patient flow. As the outbreak moves into low- and middle-income countries (LMICs), the circumstances are even more dire.^2,3^ As many of these countries have weak health systems with insufficient investment in healthcare safety, practicing proper infection prevention and control (IPC) measures is challenging because of issues such as inadequate supplies of personal protective equipment (PPE) and overcrowding. However, caring adequately for COVID-19 patients and keeping healthcare workers safe are further compromised because of a simple, but often neglected, factor within low-resource healthcare facilities: a lack of water.

According to a recent WHO/UNICEF Joint Monitoring Programme report on the status of water, sanitation, and hygiene (WASH) in healthcare facilities, one in four healthcare facilities globally lacks a functional water source on premises (i.e., basic water service), impacting nearly 2 billion people; in sub-Saharan Africa, half of all healthcare facilities have no basic water services. Furthermore, approximately 40% of healthcare facilities have no soap and water or alcohol-based hand rub at points of care.^4^

Although these data are disturbing, they do not fully reflect the day-to-day challenges of limited water supply that many healthcare providers face in LMIC health centers and hospitals. Global monitoring cannot provide a more nuanced insight into issues such as seasonal or temporary water shortages, nonfunctional water infrastructure, or fluctuating water quality, all of which impact the consistency of safe water availability and the practice of proper IPC. Researchers from the Center for Global Safe WASH at Emory University examined WASH conditions in more than 500 healthcare facilities across seven low-income countries. Our team discovered that water available today is not necessarily available tomorrow—or even 3 hours from now.

Before COVID-19, the implications of limited water access in healthcare facilities included issues of inadequate standard precautions such as poor hand hygiene and environmental cleanliness, as well as possible increases in healthcare-associated infections.^5^ In the midst of a pandemic, these concerns have intensified in low-resource healthcare facilities within LMICs, where according to the WHO, the inability to decontaminate hands and surfaces will lead to greater transmission of SARS-CoV-2, which in turn will cause serious illness and loss of life among patients and medical personnel.^6–8^ We are already acutely aware of the high rates of COVID-19–related morbidity and mortality among frontline health providers in high-income countries who struggle to maintain adequate IPC.^9–11^ The situation is even worse for medical personnel in low-resource healthcare facilities. According to the WHO Regional Office for Africa, more than 10,000 healthcare workers have contracted COVID-19 as of July 2020 and “many health centres were found to lack the infrastructure necessary to implement key infection prevention measures, or to prevent overcrowding.”^2^ This quotation from one of our IPC colleagues in Cameroon illustrates the situation firsthand: “As you already know, we have a very weak health system that is not prepared to deal with massive outbreaks like this. Water is not available, PPE is lacking...our health facilities do not have IPC/WASH structures and services...Most of the facilities do not have water, and in places where water exist, supply is erratic. Some facilities can go for days or weeks without water, and [the water] does not meet quality” (J. Gobte, personal communication).

So how did we get here? After years of research, we surmise that the norm of poor investment in WASH infrastructure and services in healthcare facilities, largely due to concerns about cost and sustainability, has led us to this situation. COVID-19 forces us to examine what happens when the prioritization of investment focuses solely on medication supply chain, and personnel and other healthcare delivery costs. By overlooking the WASH status of these healthcare facilities, we have made the assumption that adequate health care and the protection of public health lie only on the side of treatment, not prevention.

Then, what can we do? First, a change in our approach to WASH as a pillar of quality health care is needed. Health systems’ thinking has incorporated WASH as an important building block within the quality of care framework.^12^ However, in practice, WASH is not prioritized. A fundamental flaw in the delivery of health care within low-resource facilities is that we “work around” deficiencies in water access, water quality, proper sanitation, and hand hygiene facilities. Although we applaud the heroic efforts of the frontline health workers, supportive non-governmental organizations, and healthcare leaders who work in low-resource facilities, we must also demand that these healthcare facilities are equipped with the essentials. Water, sanitation, and hygiene services must be seen as nonnegotiable for a healthcare facility. Speaking at last year’s World Health Assembly, the WHO Director-General Dr. Tedros Adhanom Ghebreyesus commented that if healthcare facilities “can’t do the basics (such as WASH), then forget the rest” (T. Ghebreyesus, personal communication). We agree.

Second, solving this crisis requires clear, focused leadership from the health sector, with close collaboration from the WASH sector and other key stakeholders who play a role in ensuring sustainable WASH services in healthcare facilities.^13^ In 2018, the United Nations Secretary-General António Guterres issued a call to action to all UN agencies on the deplorable state of WASH in healthcare facilities.^14^ Subsequently, member states of the WHO demonstrated their commitment when they unanimously adopted the resolution on WASH in healthcare facilities at the 72nd World Health Assembly in May 2019.^15^ What is needed now is for national health and WASH stakeholders, including both clinicians and public health leaders, to outline country-level strategies, policies, and budgets to prioritize this essential aspect of healthcare delivery.

Third, incremental improvements to WASH services in healthcare facilities can be initiated immediately in LMICs, with the goal of both mitigating this current threat while also strengthening the health system for the future. We need to undertake WASH assessments to identify needs and prioritize improvements, such as adding portable handwashing stations to points of care and increasing water storage.^16^ We must ensure that WASH is a part of IPC training and expand such training to include nonclinical staff, such as health facility cleaners. In addition, facilities need to have empowered leadership, inclusive IPC committees that recognize WASH as fundamental to IPC, WASH champions among the staff, and accountability to local and national authorities. Many of these immediate response activities can be financed through COVID-19 funding, with the ultimate goal of transitioning to system strengthening and resiliency building, as discussed earlier.

Ultimately, what does COVID-19 reveal?—the same thing that we have always known. Over a century ago, pioneers like Dr. Ignaz Semmelweis knew that handwashing was the cornerstone of prevention against the spread of infection in healthcare settings, where an abundance of pathogens and vulnerable patients coexisted. The same is true today. So, before we narrow our priorities to innovative technologies and treatments alone, we must make sure that the tried and true methods of prevention are available in every healthcare facility, everywhere. Because in some situations, having a functional water tap is the first innovation we need.
